# Boosting BCG vaccination with MVA85A down-regulates the immunoregulatory cytokine TGF-β1

**DOI:** 10.1016/j.vaccine.2008.07.040

**Published:** 2008-09-26

**Authors:** Helen A. Fletcher, Ansar A. Pathan, Tamara K. Berthoud, Susanna J. Dunachie, Kathryn T. Whelan, Nicola C. Alder, Clare R. Sander, Adrian V.S. Hill, Helen McShane

**Affiliations:** a Centre for Clinical Vaccinology and Tropical Medicine, University of Oxford, Churchill Hospital, Oxford OX3 7LJ, UK; b Centre for Statistics in Medicine, Wolfson College, University of Oxford, Linton Road, Oxford OX2 6UD, UK

**Keywords:** TGF-β1, Regulatory T cells, Tuberculosis

## Abstract

In clinical trials recombinant-modified vaccinia virus Ankara expressing the *Mycobacterium tuberculosis* antigen 85A (MVA85A) induces approximately 10 times more effector T cells than any other recombinant MVA vaccine. We have found that in BCG primed subjects MVA85A vaccination reduces transforming growth factor beta 1 (TGF-β1) mRNA in peripheral blood lymphocytes and reduces TGF-β1 protein in the serum, but increases IFN-γ ELISPOT responses to the recall antigen SK/SD. TGF-β1 is essential for the generation of regulatory T cells and we see a correlation across vaccinees between CD4+CD25hiFoxP3+ cells and TGF-β1 serum levels. This apparent ability to counteract regulatory T cell effects suggests a potential use of MVA85A as an adjuvant for less immunogenic vaccines.

## 1 Introduction

Currently, the only tuberculosis (TB) vaccine available is BCG, which although largely ineffective at protecting against adult pulmonary disease does confer some protection against severe TB in childhood and against leprosy [1]. Therefore, strategies for improving BCG efficacy are required. Boosting BCG primed T cell responses with a second vaccine encoding an antigen present within BCG (heterologous prime–boost) is a potential strategy for improving BCG efficacy. Modified vaccinia virus Ankara (MVA) is commonly used as a vector to deliver the antigen in heterologous prime–boost strategies and has been useful in boosting T cell responses to intracellular pathogens such as HIV, malaria and hepatitis C [2–8]. Antigen 85A is considered a leading candidate antigen for inclusion in a booster vaccine for BCG. It is immunodominant in animal and human studies, is highly conserved amongst all mycobacterial species and is present in all strains of BCG. Using a recombinant MVA expressing antigen 85A (MVA85A) to boost previous BCG vaccination in BALB/c mice induces higher levels of antigen-specific IFN-γ secreting T cells and higher levels of protection from *Mycobacterium tuberculosis* (*M. tb*) challenge than BCG alone [9,10]. We have been investigating the safety and immunogenicity of MVA85A in BCG naïve and BCG primed subjects in the UK [11,12]. In these studies, MVA85A has been found to induce levels of IFN-γ secreting effector T cells approximately 10 times greater than those obtained with any other MVA vaccine construct. The effects of MVA85A vaccination on T cell regulatory mechanisms that typically limit effector T cell responses have not been previously investigated. FoxP3 is a master gene governing the development and function of regulatory T cells. Expression of FoxP3 in transgenic mice and ectopic expression of FoxP3 in human cells has been shown to genetically reprogram T cells to a regulatory phenotype [13,14]. Naturally occurring regulatory T cells are generated in the thymus are CD4+CD25high and constitute 1–5% of circulating CD4+ cells. Transforming growth factor beta 1 (TGF-β1) is ubiquitously present in a wide variety of cells and has opposing effects on the differentiation and proliferation of multiple immune-cell types [15,16]. T cells carry the TGF-β1RI and TGF-β1RII receptors and can be directly influenced through binding of active TGF-β1. TGF-β1 is also a key regulator of the signaling pathways that initiate and maintain FoxP3 expression and is essential for the generation and suppressive function of peripheral regulatory T cells [13,17,18]. Previously we have found that TGF-β1 is associated with the generation of regulatory T cells and higher rates of parasitic growth in subjects infected with *Plasmodium falciparum* [19]. The aim of this current study was to determine the effect of vaccination with MVA85A, both in BCG naïve and BCG primed subjects, on the immunoregulatory cytokine TGF-β1 and on the generation of regulatory CD4+CD25hiFoxP3+ T cells.

## 2 Materials and methods

### 2.1 Clinical trials

Subjects were recruited for immunisation studies under protocols approved by the Medicines and Healthcare products Regulatory Agency and Oxfordshire Research Ethics Committee and enrolled only after obtaining written informed consent. www.clinicaltrials.gov identifiers: NCT00480688, NCT00423566, NCT00480714 and NCT00427830. They were aged 18–55 and were all seronegative for HIV, HBV and HCV at screening. Routine laboratory haematology and biochemistry were performed prior to vaccination and all values were within normal limits. Subjects vaccinated with either BCG alone or MVA85A alone had no history of previous BCG vaccination, no evidence of BCG scar, were ELISPOT negative for the *M. tb*-specific antigens ESAT-6 and CFP-10 and had PPD responses on ELISPOT ranging from 0 to 68 (median 22) SFC per 10^6^ PBMC. Subjects vaccinated with BCG followed by MVA85A had received BCG vaccination 10–20 years prior to screening, had a visible BCG scar, were ELISPOT negative for the *M. tb*-specific antigens ESAT-6 and CFP-10 and had PPD ELISPOT responses ranging from 0 to 465 (median 177) SFC per 10^6^ PBMC. All subjects were followed-up for 6 months, with blood samples taken at regular time points. Two subjects had previously been vaccinated with vaccinia, although none had received a vaccination with MVA. Pre-existing immunity to vaccinia did not appear to effect the immune response to MVA85A.

### 2.2 IFN-γ ELISPOT assay

The main immunological measure used to determine vaccine immunogenicity was the *ex vivo* IFN-γ ELISPOT. This was performed on freshly isolated PBMC taken at the following time points: at the day of vaccination (0), and then at 1, 2, 4, 8, 12, 16, 24 and 52 weeks after vaccination as previously described [11]. These measurements were carried out on fresh PBMCs using 7 pools of 9–10 15-mer peptides, overlapping by 10 amino acids (10 μg/ml). Briefly, 300,000 PBMCs per well were plated directly onto the ELISPOT plate (MAIP, Millipore) in the presence of peptide (7 pools of 9–10 15-mer peptides overlapping by 10 amino acids, each pool final concentration 10 μg/ml), Ag85A (10 μg/ml) and PPD (20 μg/ml), and incubated for 18 h. SK/SD (streptokinase–streptodornase) (250 units and 60 units/ml) and PHA 1 μg/ml were used in all assays as positive controls. Assays were performed in duplicate and the results were averaged.

### 2.3 Cryopreservation of PBMC

PBMC were frozen in 1 ml aliquots at a concentration of 5 × 10^6^ PBMC/ml. PBMC to be frozen were centrifuged and re-suspended in 0.5 ml fetal bovine serum (FBS). A 0.5 ml aliquot of freezing mix was then added to each tube (20% dimethyl sulfoxide, 50% FBS in RPMI). Cells were immediately transferred to a Nalgene “Mr. Frosty” freezing container and stored overnight at −80 °C. Once frozen cells were transferred for long-term storage in liquid nitrogen.

### 2.4 Cell stimulation for mRNA analysis

Frozen PBMC from weeks 0, 1, 4 and 12 from six BCG, eight MVA85A and eight BCG prime–MVA85A boost vaccinees were thawed, washed and counted. 1 × 10^6^ cells in R10 (RPMI 1640 medium supplemented with 10% FBS, 2 mM l-glutamine, 100 IU/ml of penicillin and 100 IU/ml streptomycin) were plated in duplicate in 96-well plates. One pool of 66 overlapping peptides for Ag85A (15-mers overlapping by 10) was added to one duplicate well to a final concentration of 2 μg/ml each peptide. Antigen 85A peptide stimulated and media only control wells were incubated for 12 h at 37 °C 5% CO_2_. After incubation media was removed and cells lysed in 350 μl RLT buffer (RNeasy Minikit, Qiagen). Lysed cells in RLT buffer were stored at −20 °C until extraction. For IFN-γ blocking assays, media was supplemented with 10 μg/ml anti-human IFN-γ antibodies (Becton Dickinson).

### 2.5 Real-time reverse transcriptase (RT) PCR

The Rneasy Minikit (Qiagen) was used according to the manufacturers’ instructions for the extraction of total RNA from 85A peptide stimulated and media only control PBMC from each sample. Ten microliters of RNA extract was reverse transcribed in a total reaction volume of 20 μl using oligo-dT and the Omniscript Kit (Qiagen). cDNA was stored at −20 °C until use. Real-time PCR was performed using the Roche LightCycler^®^ and Quantitect mastermix (Qiagen).

Quantified, purified and diluted PCR product was used to generate external standard curves for each primer pair. *C*_t_ values were converted to copy number using these curves post-amplification. Primers were designed for HPRT (F: 5′-TATGGACAGGACTGAACGTC-3′ and R: 5′-CTACAATGTGATGGCCTCCC-3′), FoxP3 (F: 5′-CACTTACAGGCACTCCTCCAGG-3′ and R: 5′-CCACCGTTGAGAGCTGGTGCAT-3′) and TGF-β1 (F: 5′-GGACATCAACGGGTTCACTA-3′ and R: 5′-CCGGTTCATGCCATGAATGG-3′). One microliter of cDNA was used in each reaction. Cycling conditions of an initial activation step of 15 min at 95 °C followed by 45 cycles of 15 s at 94 °C, 20 s at 60 °C and 15 s at 72 °C were used for each primer pair.

### 2.6 TGF-β1 ELISA

Serum from weeks 0, 1, 4 and 12 from 6 BCG, 8 MVA85A and 11 BCG prime–MVA85A boost vaccinees were analysed for TGF-β1 protein using the human TGF-β1 ELISA kit from eBioscience (San Diego, CA). Briefly, serum was diluted 1/5 with PBS and 10 μl 1N HCl added to 100 μl of diluted sample. After 10 min incubation samples were neutralised with 10 μl 1N NaOH and transferred to a pre-coated plate. Following an over night incubation at 4 °C the colour was developed according to the manufacturers instructions Plates were read at 450 nm/570 nm in a MultiSkan (Thermo Life Sciences) plate reader.

### 2.7 Flow cytometry

FoxP3 mRNA expression was confirmed using intracellular staining for FoxP3 protein. The anti-human Foxp3 antibody (PCH101 clone), CD4 FITC (RPA-T4), CD25 APC (BC96) were purchased from eBioscience as part of the Human Regulatory T Cell Staining Kit (San Diego, CA). Intracellular staining for FoxP3 was performed on PBMC from subjects vaccinated with MVA85A alone according to the manufacturers’ instructions. FACSCalibur (Becton Dickinson) was used for flow cytometry event collection and events were analysed using FlowJo (Tree Star Inc.).

### 2.8 Statistical analysis

For the real-time RT-PCR data the mean *C*_t_ value of duplicate capillaries was converted to copy number using external standard curves generated from purified and quantified PCR product. TGF-β1 and FoxP3 expression were normalised by dividing copy number of gene by copy number of HPRT. Normalised mRNA and serum protein values within a vaccine group were compared using the non-parametric Wilcoxon Signed Rank test in SPSS. Correlations between TGF-β1 protein, IFN-γ ELISPOT responses and CD4+CD25hiFoxP3+ cells were performed using the Spearman's Rank test in SPSS. Area under the curve analysis was carried out and the three groups were compared using the Kruskall–Wallis test. Where significant differences were found between the three groups, the Mann–Whitney test was used to make all pair-wise comparisons. These analyses were carried out using STATA.

## 3 Results

### 3.1 TGF-β1 but not FoxP3 mRNA expression is reduced following vaccination with MVA85A in BCG primed subjects

The kinetics of FoxP3 mRNA and TGF-β1 mRNA expression were investigated in BCG naïve or BCG primed subjects following vaccination with MVA85A. mRNA was measured on the day of vaccination and 1, 4 and 12 weeks following vaccination. An area under the curve analysis (AUC) was carried out between 0 and 12 weeks to determine differences in expression between the vaccine groups. A significant difference in TGF-β1 mRNA expression between the vaccine groups was seen in PBMC stimulated with 85A peptides (*p* = 0.03) and a weak difference was seen in unstimulated PBMC (*p* = 0.06) (Table 1). There was no significant difference in FoxP3 mRNA expression between the vaccine groups. A pair-wise test of TGF-β1 mRNA expression using Mann–Whitney showed a significant decrease in both stimulated and unstimulated TGF-β1 mRNA expression in BCG primed versus BCG naïve subjects vaccinated with MVA85A (*p* = 0.02, 0.03) (Table 2).

### 3.2 TGF-β1 protein is reduced in serum following vaccination with MVA85A in BCG primed subjects

**Fig. 1:**
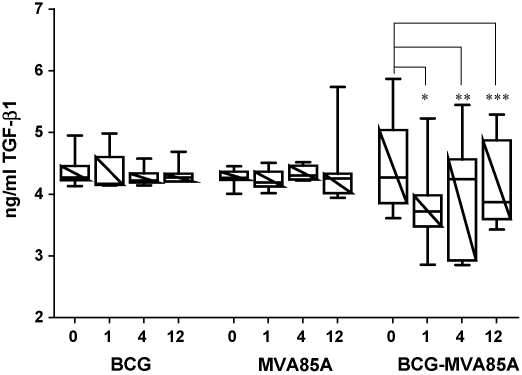
Serum levels of TGF-β1 protein are decreased in BCG primed subjects following boosting with MVA85A but not in subjects vaccinated with BCG alone or MVA85A alone. Serum TGF-β1 protein levels were measured on the day of vaccination and 1, 4 and 12 weeks post-vaccination for subjects vaccinated with BCG alone (*n* = 6), MVA85A alone (*n* = 8) and in BCG primed subjects vaccinated with MVA85A (*n* = 11). There was a significant decrease in serum TGF-β1 protein in BCG primed subjects following boosting with MVA85A, *p = 0.0005, **p = 0.021, ***p = 0.021 (Wilcoxon). Box and whisker plots showing median, 25th and 75th percentiles.

TGF-β1 mRNA was significantly reduced in both peptide-stimulated and -unstimulated PBMC from BCG primed subjects vaccinated with MVA85A. TGF-β1 protein is secreted as a latent proprotein which can be activated by acidification in vitro. Acid activated serum TGF-β1 protein levels were measured pre- and post-vaccination to determine if the reduction in TGF-β1 mRNA reflected a systemic reduction in TGF-β1 protein. There was a significant decrease in serum TGF-β1 from day of vaccination (median 4.3 ng/ml) to week 1 (median 3.7 ng/ml), at week 4 TGF-β1 increased but remained significantly below the day of vaccination (median 4.2 ng/ml) and by week 12 TGF-β1 levels had decreased again (median 3.9 ng/ml) (Fig. 1). Following vaccination with either BCG alone or MVA85A alone there was no significant change in serum TGF-β1 protein (Fig. 1). Although both TGF-β1 mRNA and protein were reduced following vaccination of BCG primed subjects with MVA85A there was no direct correlation of acid activated TGF-β1 protein and mRNA expression (*p* = 0.294). Others have reported a lack of direct correlation between TGF-β1 mRNA and protein ([38], #130). This is most likely due to the secretion of TGF-β1 as a latent proprotein requiring proteolytic cleavage of the LAP peptide (latency associated propeptide). TGF-β1 is also produced by many cell types, not just PBMC, which may account for the lack of direct correlation between TGF-β1 protein and mRNA in our study.

### 3.3 Higher serum TGF-β1 protein on the day of vaccination results in lower IFN-γ ELISPOT responses to Ag85A peptide up to 4 months following vaccination with MVA85A alone

To determine if TGF-β1 protein at week 0 could effect long-term memory responses serum TGF-β1 was correlated (Spearman's correlation) with IFN-γ ELISPOT responses following vaccination with BCG alone, MVA85A alone and BCG primed subjects vaccinated with MVA85A. There was no relationship between serum TGF-β1 and peptide responses to BCG at any time point. TGF-β1 protein at week 0 inversely correlated with IFN-γ ELISPOT responses at weeks 4, 8, 12 and 16 following vaccination with MVA85A. The relationship was lost at weeks 24 following vaccination with MVA85A, however, the peptide IFN-γ ELISPOT response was undetectable (0 spots) in many volunteers at this time point which may account for the lack of correlation with TGF-β1. In contrast, when MVA85A is administered to BCG primed subjects there is little correlation with TGF-β1 protein and IFN-γ ELISPOT responses at the earlier time points. Presumably because TGF-β1 is reduced in BCG primed subjects vaccinated with MVA85A. However, there is a trend towards significance at the later time points (weeks 24 and 52) (Table 3).

### 3.4 Immune responses to the recall antigen SK/SD are increased in BCG primed subjects following vaccination with MVA85A

**Fig. 2:**
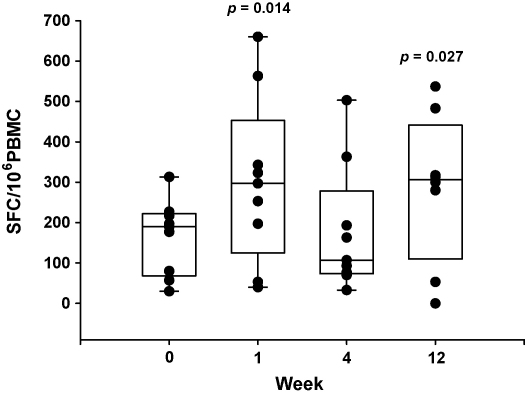
Immune responses to the recall antigen SK/SD are increased at both weeks 1 and 12 in BCG primed subjects vaccinated with MVA85A (*n* = 9) (Wilcoxon). Box and whisker plots showing median, 25th and 75th percentiles.

To determine if the reduction in TGF-β1 serum protein could lead to a non-specific enhancement of effector T cell responses in vaccinated subjects IFN-γ ELISPOT responses to the recall antigen SK/SD were determined at weeks 0, 1, 4 and 12. SK/SD responses were significantly enhanced in BCG primed subjects at week 1 (median = 292 SFC/million) returned to baseline at week 4 (median = 107 SFC/million) and were enhanced again at week 12 post-vaccination with MVA85A (median = 307 SFC/million) (Fig. 2). This bi-modal increase in the SK/SD ELISPOT response seems to be the inverse of that seen with TGF-β1 protein in the serum. SK/SD responses were unchanged following vaccination with either BCG alone or MVA85A alone (data not shown).

### 3.5 SK/SD recall responses in BCG primed subjects are inversely proportional to TGF-β1 serum protein levels

**Fig. 3:**
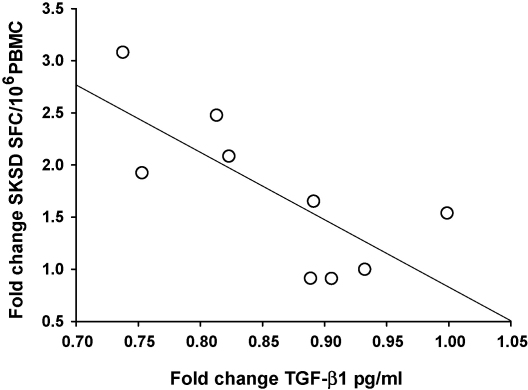
The immune response to the recall antigen SK/SD (fold change of week 1 over week 0) is inversely proportional to TGF-β1 serum protein levels (fold change of week 1 over week 0). *n* = 9, *R* = −0.769, *p* = 0.008 (Spearman's correlation).

Spearman's correlation was performed to determine if the increase in immune response to recall antigen could be related to the decrease in TGF-β1 mRNA or serum protein. The SK/SD IFN-γ ELISPOT responses at week 1 were significantly inversely correlated to TGF-β1 serum protein but not TGF-β1 mRNA in BCG primed subjects (*p* = 0.008) (Fig. 3).

### 3.6 Inverse correlation of CD4+FoxP3+ CD25^high^ lymphocytes with TGF-β1 serum protein and SK/SD IFN-γ ELISPOT responses

**Fig. 4:**
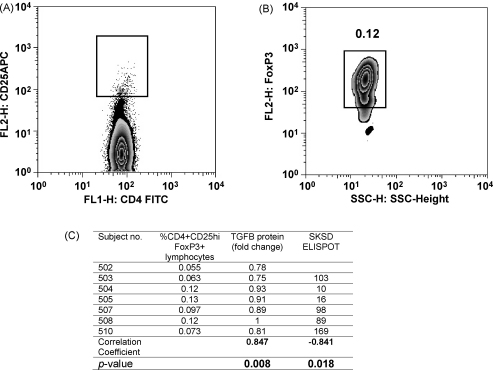
The percentage of CD4+CD25hiFoxP3+ T cells 1 week post-vaccination of BCG primed subjects with MVA85A are inversely proportional to the SK/SD ELISPOT response. The percentage of total CD4+CD25 high FoxP3 expressing cells in the lymphocyte gate was determined by (A) gating on CD4 positive CD25high cells. (B) FoxP3 expressing cells were selected from within the CD4+CD25high gate. Representative plots from 1 of 6 subjects are shown. (C) Spearman's correlation of the percentage of CD4+CD25hiFoxP3+ T cells with fold change in TGF-β1 protein expression and SK/SD ELISPOT response.

TGF-β1 can either directly regulate effector T cells or indirectly regulate effector T cells through the activity of regulatory T cells. CD4+CD25hi cells express high levels of FoxP3 and are a highly efficient regulatory subset of the CD25+ population [20]. The percentage of CD4+CD25hiFoxP3+ cells, 1 week following vaccination with MVA85A, in the PBMC of six BCG primed subjects vaccinated with MVA85A were determined using flow cytometry. To determine where the CD25hi population lay, a dot plot showing the distribution of CD25 versus CD4 was used (Fig. 4A). FoxP3+ lymphocytes were selected within the CD4+CD25hi gate (Fig. 4B). The percentage of CD4+CD25hiFoxP3+ cells in PBMC inversely correlated with SK/SD IFN-γ ELISPOT responses in BCG primed subjects vaccinated with MVA85A (*p* = 0.018, Fig. 4C). The percentage of CD4+CD25hiFoxP3+ cells 1 week post-vaccination positively correlated with TGF-β1 protein in the serum of BCG primed subjects (*p* = 0.008, Fig. 4C). There was no correlation between CD4+CD25hiFoxP3+ cells with IFN-γ ELISPOT responses to 85A peptides or PPD (data not shown).

### 3.7 Treatment of PBMC with anti-IFN-γ antibodies increases the expression of TGF-β1 mRNA

**Fig. 5:**
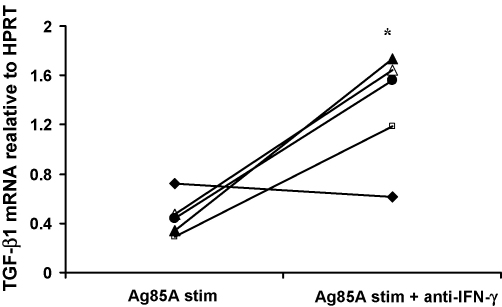
Treatment of PBMC with anti-IFN-γ antibodies increases the expression of TGF-β1 mRNA. IFN-γ can lead to a down-regulation in TGF-β1 signaling. Blocking IFN-γ leads to a significant increase in TGF-β1 mRNA expression in BCG primed subjects 4 weeks post-vaccination with MVA85A, *p = 0.016 (Wilcoxon).

A possible mechanism for the reduction in TGF-β1 mRNA in the BCG primed group is that the higher level of antigen-specific IFN-γ in the BCG prime–MVA85A boost group favors the down-regulation of TGF-β1 signaling and mRNA production. Cells from five BCG primed subjects vaccinated with MVA85A were cultured with 85A peptides and IFN-γ blocking antibodies. Blocking IFN-γ gamma significantly increased TGF-β1 mRNA expression in these subjects (*p* = 0.016) (Fig. 5).

## 4 Discussion

The vaccination of BCG primed subjects with MVA85A induces levels of IFN-γ secreting effector T cells approximately 10 times greater than those reported for any other recombinant MVA [3,4,6–8,21–23]. BCG vaccination induces Ag85A-specific memory T cells and Ag85A is also present in many environmental mycobacteria. MVA85A therefore has a potentially large pool of Ag85A-specific memory T cells that could be expanded into IFN-γ secreting effector T cells. In this current study we have also found that vaccination with MVA85A in BCG primed but not BCG naïve subjects leads to a reduction in serum TGF-β1 protein. Although we see no direct correlation between protein and mRNA, the reduction in TGF-β1 serum protein is likely to be controlled at the transcriptional level as we see a reduction in TGF-β1 mRNA in the PBMC of vaccinated subjects. TGF-β1 is a key regulator of the signaling pathways that initiate and maintain FoxP3 expression and is essential for the generation and suppressive function of peripheral regulatory T cells [13,17,18]. However, we saw a transient increase rather than a decrease in the expression of FoxP3 mRNA. It is known that FoxP3 mRNA is transiently expressed by T cells upon activation and that this does not commit cells to a regulatory phenotype [24]. The changes in FoxP3 mRNA expression we observe in response to MVA85A vaccination are likely to be a result of T cell activation rather than T cell regulation.

Previously we have found that TGF-β1 is associated with the generation of regulatory T cells and higher rates of parasitic growth in subjects infected with *P. falciparum* [19]. In murine studies, we and others recently found that depletion of regulatory T cells using a monoclonal antibody to CD25 enhances the immunogenicity of a variety of vaccine types including BCG and MVA85A [25,26]. In our current study the decrease in TGF-β1 serum protein inversely correlated with an increase in IFN-γ ELISPOT response to the recall antigen SK/SD. The effect appears to be bi-modal with both TGF-β1 and SK/SD responses returning close to baseline at week 4 before becoming reduced/increased again at week 12. We found that both TGF-β1 serum protein and SK/SD IFN-γ ELISPOT responses correlated with the percentage of CD4+CD25hiFoxP3+ T cells in BCG primed subjects. The increase in ELISPOT response to recall antigen may therefore be a result of systemic reduction in TGF-β1 protein and subsequent reduction in the number of peripheral regulatory CD4+CD25hiFoxP3+ T cells. Alternatively, T cells can be directly regulated through binding of active TGF-β1 with the TGF-β1RI and TGF-β1RII cell surface receptors. It has recently been found that serum TGF-β1 is the most prominent factor actively keeping CD4+ T cells in a resting state [27]. Classens et al. found that the removal of even low physiological concentrations of TGF-β1 had a functional effect on CD4+ T cells, leading to enhanced proliferation in response to low level TCR signaling. Interestingly, despite the capacity of viral vectors to boost a CD8 response, the antigen-specific response following vaccination with MVA85A has been exclusively a CD4+ response (when measured by IFN-γ ELISPOT) [11,12]. This may be due to enhanced activation and proliferation of CD4+ T cells in response to lowered TGF-β1 serum concentrations. High TGF-β1 serum protein appears to correlate with reduced effector T cell responses to MVA85A in BCG naïve subjects. However, in BCG primed subjects effector T cell responses are higher and there is no association with TGF-β1. Down-regulation of TGF-β1 is likely, at least in part, to account for the sustained high levels of antigen-specific effector T cells seen after vaccination with MVA85A in BCG primed subjects, perhaps through a reduction in the number of circulating regulatory T cells.

Plasma TGF-β1 levels are predominantly under genetic control [28]. However, IFN-γ can directly regulate TGF-β1 through the STAT and SMAD pathways [29]. One possible mechanism for the reduction in TGF-β1 is that the high level of antigen-specific IFN-γ released following vaccination with MVA85A leads to a down-regulation of TGF-β1 mRNA via expression of SMAD 7 [29]. Indeed, we have found that blocking IFN-γ leads to an increase in antigen-specific TGF-β1 mRNA expression. There is strong evidence for TGF-β1-dependent immune regulation in tuberculosis infection. When compared to healthy controls, TB patients have been found to have reduced IFN-γ production and increased TGF-β1 in response to stimulation with mycobacterial antigens [30–33]. Neutralising TGF-β leads to enhanced immune responses and clearance of tuberculosis infection [30,32,34]. There is also evidence for the expansion of FoxP3 expressing regulatory T cells during tuberculosis infection [35–37].

Although the number of subjects in our study is small, we have seen that vaccination of BCG primed subjects with MVA85A results in reduced TGF-β1 mRNA in PBMC and reduced TGF-β1 protein in the serum of vaccinated subjects. This appears to limit the number of circulating CD4+CD25hiFoxP3 positive T cells and enhance IFN-γ responses to the recall antigen SK/SD. Therefore, in BCG primed individuals there may be a period of time shortly following MVA85A vaccination where greater effector T cell responses could be elicited from a less immunogenic vaccine. This raises the possibility of designing vaccine schedules for TB, HIV and malaria whereby the vaccine for TB aids in the generation of protective immunity against HIV and malaria.

## Figures and Tables

**Table 1:** Following vaccination an area under the curve analysis (AUC) was carried out between 0 and 12 weeks to measure changes in FoxP3 or TGF-β1 mRNA expression

Vaccine group	*n*	Median AUC (25th, 75th percentiles)			
		FoxP3-unstimulated expression	FoxP3 85A-stimulated expression	TGFβ-unstimulated expression	TGFβ 85A-stimulated expression
BCG	5	1.34 (1.26–1.63)	1.60 (1.38–1.68)	36.81 (33.53–37.61)	35.62 (34.28–38.30)
BCG–MVA	7	1.43 (0.98–2.12)	1.18 (1.02–1.84)	27.24 (21.15–36.36)	26.65 (17.73–34.39)
MVA	8	1.21 (1.07–1.42)	1.43 (1.19–1.59)	37.84 (31.06–43.30)	37.54 (31.29–44.62)
*p*-Value^a^		0.60	0.43	0.06	0.03

The Kruskall–Wallis test (a global test for the comparison of medians) was performed to identify significant changes in gene expression. Data are summarized using median and interquartile range. Two subjects without data at 12 weeks had to be excluded from the analysis

a Kruskall–Wallis test.

**Table 2:** The expression of TGF-β1 mRNA is significantly reduced following vaccination with BCG–MVA85A when compared to BCG alone or MVA85A alone

	TGFβ-unstimulated expression		TGFβ 85A-stimulated expression	
	Diff in medians (95% CI^a^)	*p*-Value^b^	Diff in medians (95% CI^a^)	*p*-Value^b^
BCG–MVA vs. BCG	−8.81 (−16.07–2.58)	0.09	−10.32 (−25.53–0.79)	0.06
BCG–MVA vs. MVA	−9.63 (−18.74–−0.34)	0.03	−12.47 (−25.67–−2.54)	0.02
BCG vs. MVA	−1.83 (−9.86–5.92)	0.66	−2.04 (−9.93–5.73)	0.59

The Mann–Whitney test was used to determine significant differences in gene expression.

a Estimated using the Binomial method.

b Mann–Whitney test.

**Table 3:** Higher serum TGF-β1 protein on the day of vaccination results in lower IFN-γ ELISPOT responses to Ag85A peptides 4 months following vaccination with MVA85A alone

	Week 1	Week 4	Week 8	Week 12	Week 16	Week 24	Week 52
BCG							
Correlation coefficient	−0.06	0.23	−0.49	0.05	ND	−0.03	ND
*p*-Value (1 − tailed)	0.457	0.329	0.160	0.467		0.478	
*n*	6	6	6	5		6	

MVA85A							
Correlation coefficient	−0.44	−0.77	−0.85	−0.71	−0.81	0.02	ND
*p*-Value (1 − tailed)	0.136	**0.020**	**0.004**	**0.024**	**0.007**	0.484	
*n*	8	7	8	8	8	7	

BCG–MVA85A							
Correlation coefficient	−0.07	−0.10	ND	−0.16	ND	−0.53	−0.57
*p*-Value (1 − tailed)	0.416	0.385		0.315		0.059	0.090
*n*	11	11		11		10	7

To determine if TGF-β1 protein at week 0 could effect long-term memory responses serum TGF-β1 was correlated (Spearman's correlation) with IFN-γ ELISPOT responses following vaccination with BCG alone, MVA85A alone and MVA85A vaccination of BCG primed subjects.
